# Supplementary material for: Re-Arrangements in the Cytoplasmic Distribution of Small RNAs Following the Maternal-to-Zygotic Transition in Drosophila Embryos
Source: Genes (Basel). 2018 Feb 10;9(2):82. doi: 10.3390/genes9020082 (PMC5852578; doi:10.3390/genes9020082)
Supplement: Supplementary file 1 [file genes-09-00082-s001.zip › Supplementary files before conversion/Supplementary figures.docx]

Re-Arrangements in the Cytoplasmic Distribution of Small RNAs Following the Maternal-to-Zygotic Transition in *Drosophila* Embryos

Mehmet Ilyas Cosacak ^1,2,3^, Hatice Yiğit ^1,4^, Caghan Kizil^2,3^, Bünyamin Akgül ^1,^*

^1^ Department of Molecular Biology and Genetics, İzmir Institute of Technology, Gülbahçeköyü, 35430 İzmir, Turkey; mehmet.cosacak@dzne.de (M.I.C.); hatyigit@uni-mainz.de (H.Y.); caghan.kizil@dzne.de (C.K)

^2^ German Center for Neurodegenerative Diseases (DZNE) Dresden, Helmholtz Association, Arnoldstr. 18, 01307, Dresden, Germany

^3^ Center for Regenerative Therapies Dresden (CRTD), Technische Universität Dresden, Fetscherstr. 105, 01307, Dresden, Germany

^4^ Present address: Institute for Developmental Biology and Neurobiology, Johannes Gutenberg-University Mainz, Bentzelweg 3, 55128 Mainz, Germany

***** Correspondence: bunyaminakgul@iyte.edu.tr; Tel.: +011-90-232-7507316; Fax: +011-90-232-7507302

| 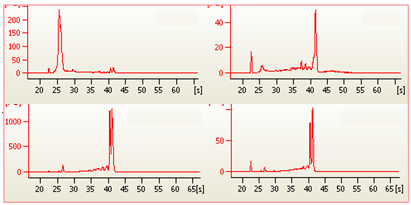 |
| --- |
| (**a**) |
| 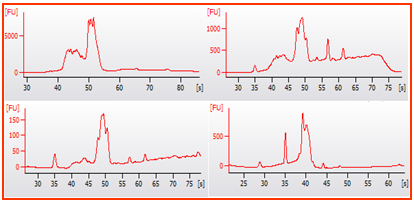 |
| (**b**) |

**Supplementary Figure 1.** Total RNAs phenol extracted from fractionated embryos were run on Bionalyzer to assess the quality and size distribution of RNAs. Electropherograms of total RNAs using Agilent RNA 6000 Nano kit (**a**) and small RNA kit (**b**).


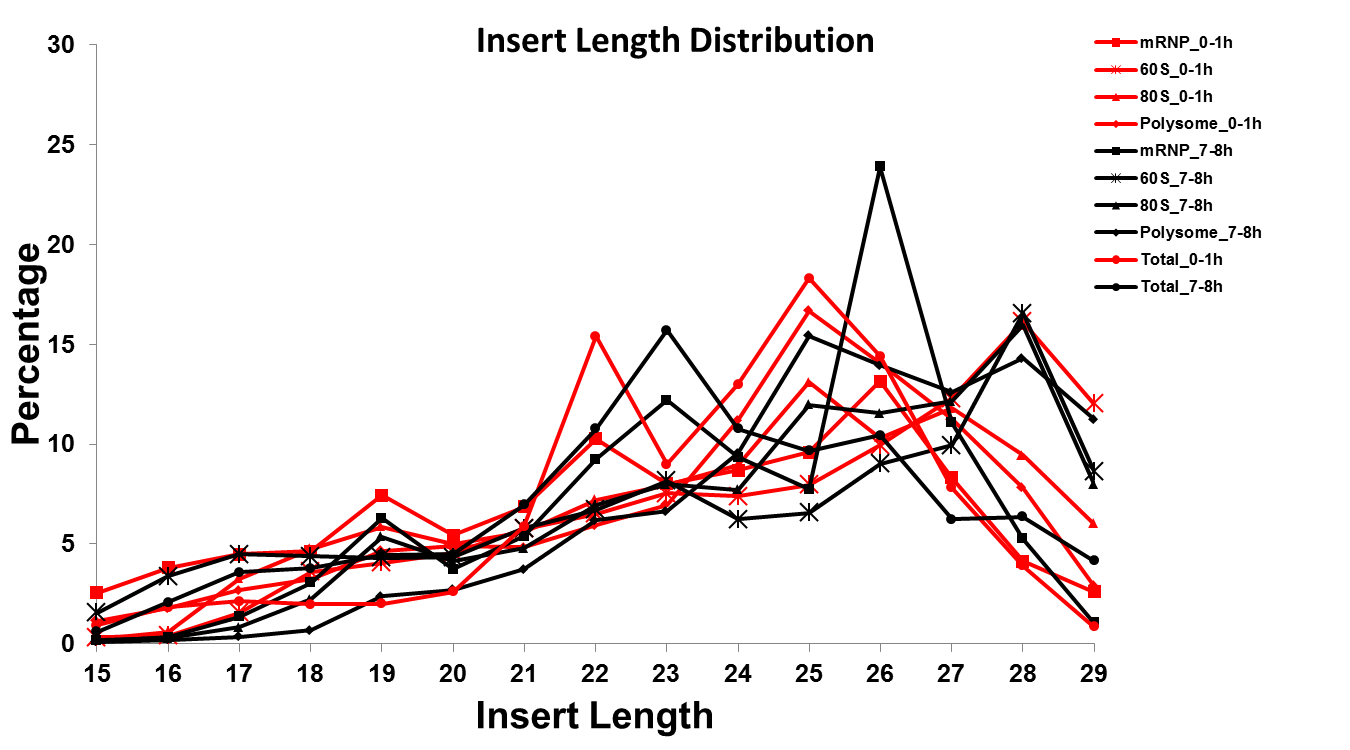


**Supplementary Figure 2.** The insert length distribution of small RNAs.


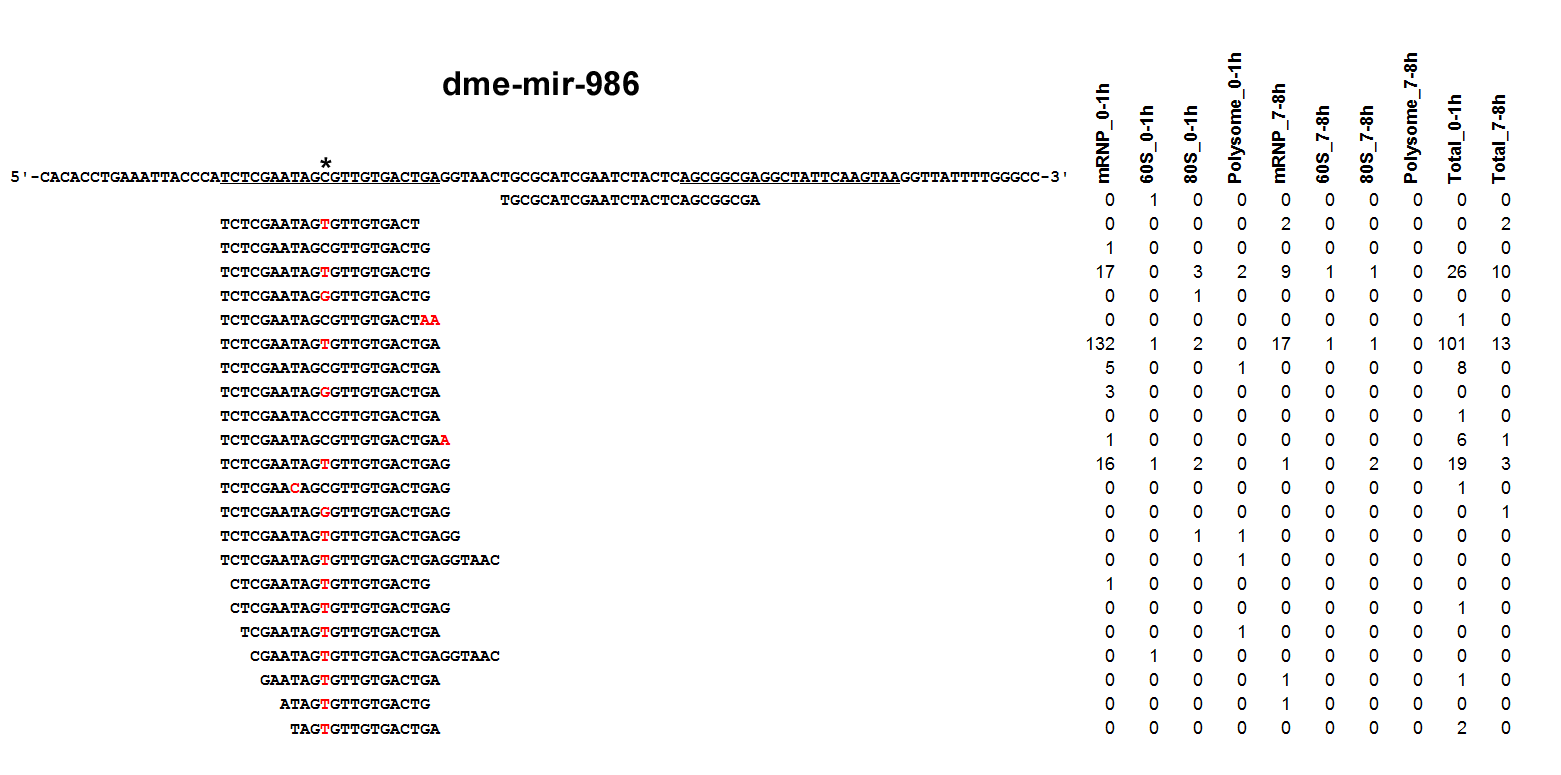


**Supplementary Figure 3.** SNP on dme-miR-986-5p. The hairpin sequence of dme-mir-986 has been shown, and dme-miR-986-5p and dme-miR-986-3p are underlined. All exact matched and single matched reads are aligned under the hairpin sequence, and nucleotide differences are red. The raw reads number are shown on the right for each read. *: SNP nucleotide.

| 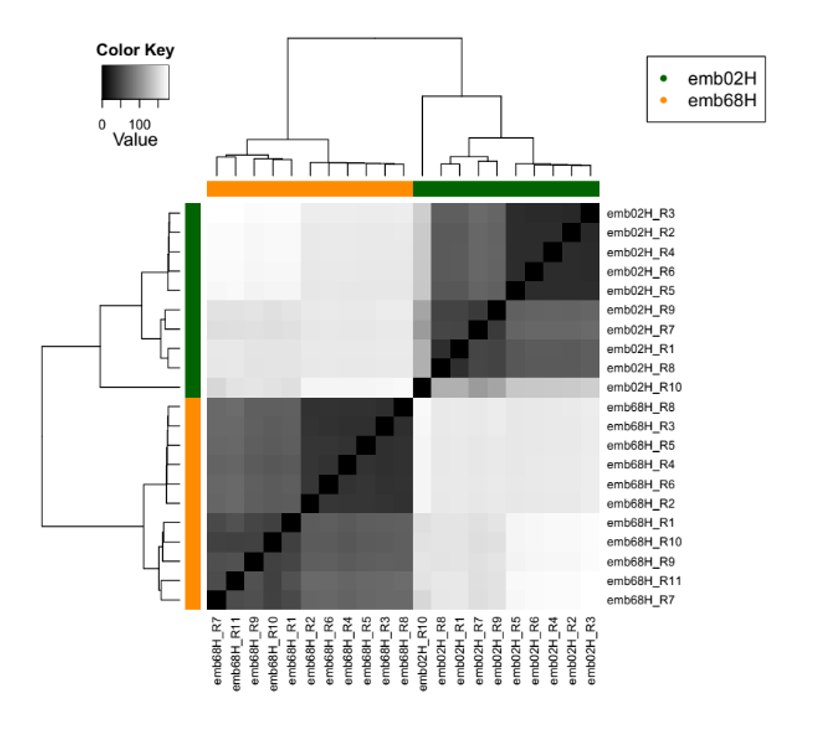 | 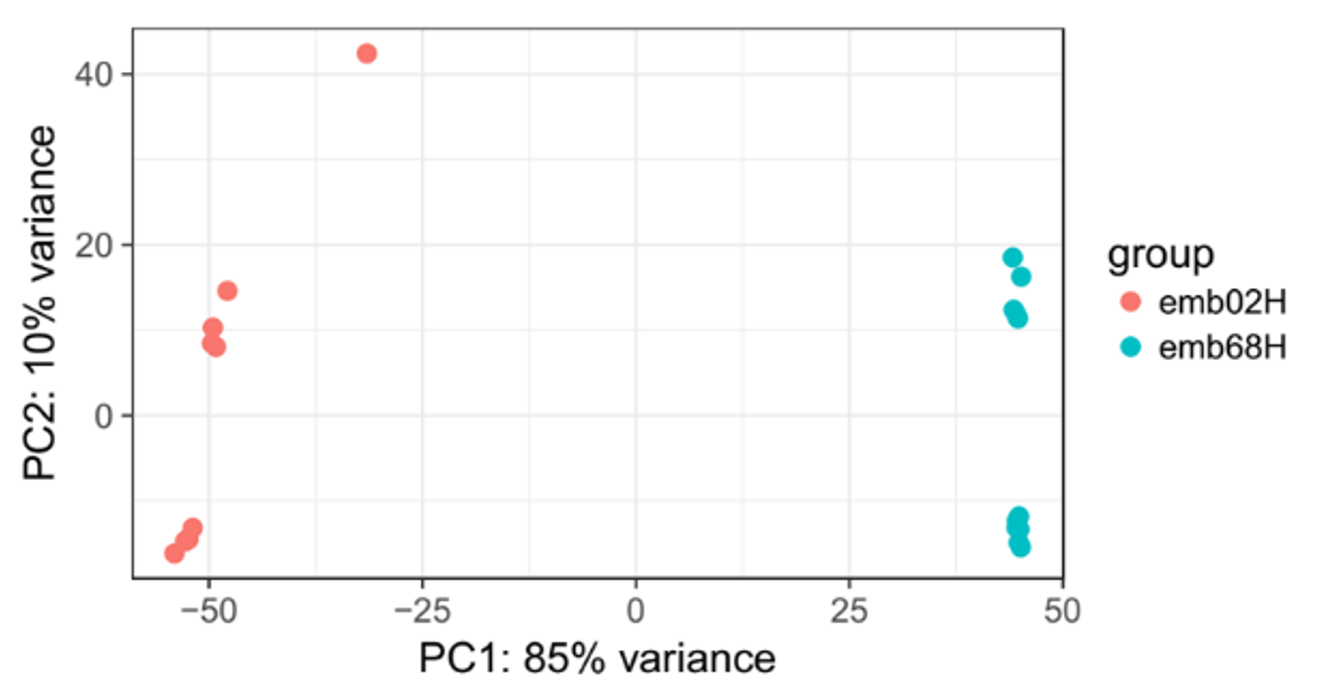 |
| --- | --- |
| (**a**) | (**b**) |
| 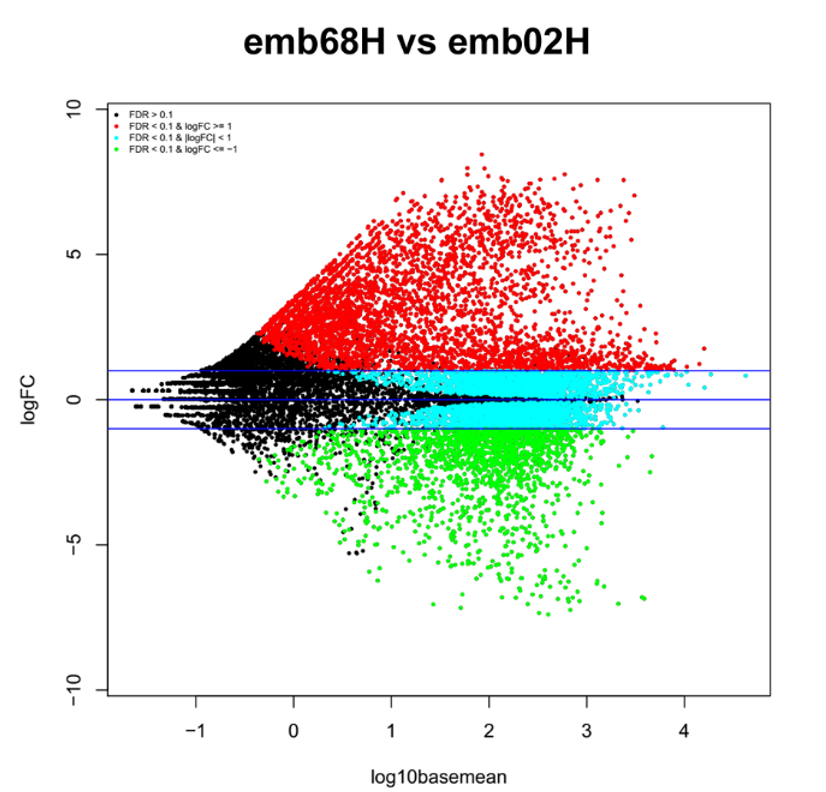 | |
| (**c**) | |

**Supplementary Figure 4.** Analysis of 0-2 and 6-8 hour old embryonic RNA-Seq data. The heat-map of distances between each datasets (A), the principle component analysis of each samples (B) and the MA-plot (C) are generated by using DESeq2 [1]. emb02H: embryo 0-2 hour, emb68H: embryo 6-8 hour, R: replicate number.


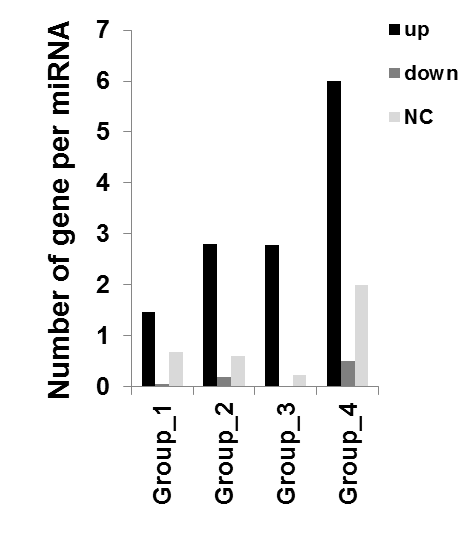


**Supplementary Figure 5.** Correlation between miRNA and their targets. We downloaded miRNA and their targets from DIANA Tarbase [2]. We then categorized the differentially expressed transcripts from modENCODE project [3] (up, up-regulated; down, down-regulated; NC, no change). Then we sorted the differentially expressed genes that are the validated targets of miRNAs in our four miRNA groups. The number of genes that were up (up-regulated), down (down-regulated) and NC (no-change) were then divided by the number of miRNAs in each group for normalization to obtain the number of differentially expressed genes per miRNA.

References

1. Love, M.I.; Huber, W.; Anders, S. Moderated estimation of fold change and dispersion for rna-seq data with deseq2. *Genome Biol* **2014**, *15*, 550.
2. Vlachos, I.S.; Zagganas, K.; Paraskevopoulou, M.D.; Georgakilas, G.; Karagkouni, D.; Vergoulis, T.; Dalamagas, T.; Hatzigeorgiou, A.G. Diana-mirpath v3.0: Deciphering microrna function with experimental support. *Nucleic Acids Res* **2015**, *43*, W460-466.
3. Celniker, S.E.; Dillon, L.A.; Gerstein, M.B.; Gunsalus, K.C.; Henikoff, S.; Karpen, G.H.; Kellis, M.; Lai, E.C.; Lieb, J.D.; MacAlpine, D.M.*, et al.* Unlocking the secrets of the genome. *Nature* **2009**, *459*, 927-930.

© 2018 by the authors. Submitted for possible open access publication under the
terms and conditions of the Creative Commons Attribution (CC BY) license (http://creativecommons.org/licenses/by/4.0/).
